# HYPERTENSION CONTROL PROGRAM IN A LARGE HEALTH CARE SYSTEM SERVING HISPANIC MEDICARE BENEFICIARIES IN SOUTH FLORIDA

**DOI:** 10.1093/geroni/igae098.2458

**Published:** 2024-12-31

**Authors:** Paulo Chaves, Maureen Desoria, Leslye Roque, Rafael Mas

**Affiliations:** Florida International University, Miami, Florida, United States; Leon Medical Centers, LLC, Miami, Florida, United States; Leon Medical Centers, LLC, Miami, Florida, United States; Leon Medical Centers, LLC, Miami, Florida, United States

## Abstract

Healthcare systems play a key role in hypertension control and health equity promotion in large populations. This study assessed the impact of a multifaceted hypertension control program in a large healthcare system serving Medicare and dual eligible (DE) Beneficiaries primarily of Hispanic origin in South Florida, an understudied population. Leon Medical Centers, a major integrated healthcare services provider to Medicare and DE patients in Miami-Dade, FL, implemented a hypertension control program in 2011. Electronic health records (2008-2018) from patients aged 65-89 years with hypertension were analyzed. Controlled BP definition was annual average systolic BP< 140 mmHg and diastolic BP< 90 mmHg. Age- and diabetes-standardized annual BP control rates were calculated. Random effects modeling compared BP control levels after vs. before program implementation. From 2008 (n=4,710) to 2018 (n=21,540), mean age±SD increased (73.4±5.3 to 77.1±6.1). Proportion of Hispanics remained constant (>98%). Overall age-adjusted odds of BP control were 5.8 (95% confidence interval [CI]: 5.6-6.1) times higher after program implementation. BP control rates increased similarly in both sexes. In women, standardized control rates increased from 68.9-73.3% (2008-2010) to 88.8-92.3% (2013-2018). Standardized control rates tended to be lower in DE than in Medicare patients at baseline – e.g., 2008: 67.2% (65.9-69.3%) vs. 72.2% (70.4-73.8%), but similar after program implementation – e.g., 2018: 89.9% (89.3-90.5%) vs. 90.2% (89.4-91.0%). Implementation of a primary care-based, multicomponent, culturally congruent hypertension control program was associated with meaningful increases in BP control levels in both men and women, and Medicare and DE patients in a large healthcare system in South Florida.

